# Case report: Two PD-L1 positive unresectable advanced pancreatic carcinoma patients with microsatellite stability achieved R0 resection after PD-1 antibody plus chemotherapy as a successful downstaging therapy: A report of two cases

**DOI:** 10.3389/fimmu.2022.946266

**Published:** 2022-09-20

**Authors:** Lin Shang, Peng Li, Jie Fan, Chunning Zhao, Xiangying Niu, Qitian Bian, Zhilin Yuan, Yanlong Kong, Tingshun Zhu, Bin Xu, Jianxin Dong, Hongjun Xiang

**Affiliations:** ^1^ Department of Hepatobiliary Surgery, Xi’an Daxing Hospital, Xi’an, China; ^2^ Department of Pathology, Xi’an Daxing Hospital, Xi’an, China

**Keywords:** unresectable advanced pancreatic cancer, downstaging therapy, chemotherapy, immunotherapy, PD-1 antibody

## Abstract

**Background:**

Nonobvious early symptoms are a prominent characteristic of pancreatic cancer, resulting in only 20% of patients having resectable tumors at the time of diagnosis. The optimal management of unresectable advanced pancreatic cancer (UAPC) remains an open research question. In this study, the tumors shrank significantly after PD-1 antibody combined with chemotherapy in two UAPC patients, and both have achieved R0 (pathologically negative margin) resection and survival to date.

**Case presentation:**

Case 1: A 53-year-old man was diagnosed with pancreatic adenocarcinoma (Stage III). He received six cycles of PD-1 antibody plus chemotherapy as the first-line treatment. The tumor was reduced from 11.8×8.8 cm to “0” (the pancreatic head was normal as shown by enhanced computed tomography, ECT) after preoperative neoadjuvant therapy (PNT) and the adverse effects were tolerable. The patient underwent radical surgery and achieved R0 resection. Case 2: A 43-year-old man diagnosed with pancreatic adenocarcinoma with liver metastasis (Stage IV) received three cycles of PD-1 antibody combined with chemotherapy. The tumor was reduced from 5.2×3.9 cm to 2.4×2.3 cm with no side effects. The patient also underwent radical surgery and achieved R0 resection.

**Conclusion:**

PD-1 antibody plus a chemotherapy regimen resulted in a surprising curative effect and safety in two patients with UAPC, which may portend an improvement in pancreatic carcinoma treatment. We may have a way for UAPC patients to obtain radical treatment and gain long-term survival. Two PD-L1 positive UAPC patients with microsatellite stability (MSS) enlighten us to have a more comprehensive understanding of the prediction of immunotherapy.

## Introduction

Pancreatic cancer is a digestive system malignant tumor with an increasing incidence rate and a high mortality. In recent years, it has become an increasingly common cause of cancer-related death. Patients with different degrees of pancreatic cancer are divided into resectable, borderline resectable, and unresectable (locally advanced, metastatic) pancreatic carcinoma (1). Poor prognosis is a typical feature of patients with pancreatic cancer, resulting in more than 80% of patients being unable to undergo radical resection surgery at the time of diagnosis (2). Recently, accumulating evidence supports the consideration of preoperative neoadjuvant therapy (PNT) when facing unresectable (potentially resectable) pancreatic cancer (3, 4). Clinical studies have reported that PNT can improve the conversion rate, decrease surgical complexity, especially in tumor volumes and vascular invasion, increase the success of radical surgery, increase the R0 resection rate, and prolong disease-free survival and overall survival in patients with UAPC (5). In 2019, a study confirmed that neoadjuvant chemotherapy plus radiation therapy could achieve a 61% R0 resection rate in subjects with locally advanced pancreatic cancer (6). However, National Comprehensive Cancer Network guidelines state that there is still a lack of clinical therapeutic regimens for neoadjuvant therapy from large-sample phase III clinical studies, and there currently are no accepted solutions. Herein we report two cases of UAPC that were successfully converted following PD-1 antibody combined with chemotherapy and in which R0 resection was achieved. Our results suggest that this PNT is a promising strategy in patients with UAPC.

## Case presentation

### Case 1

**Figure 1:**
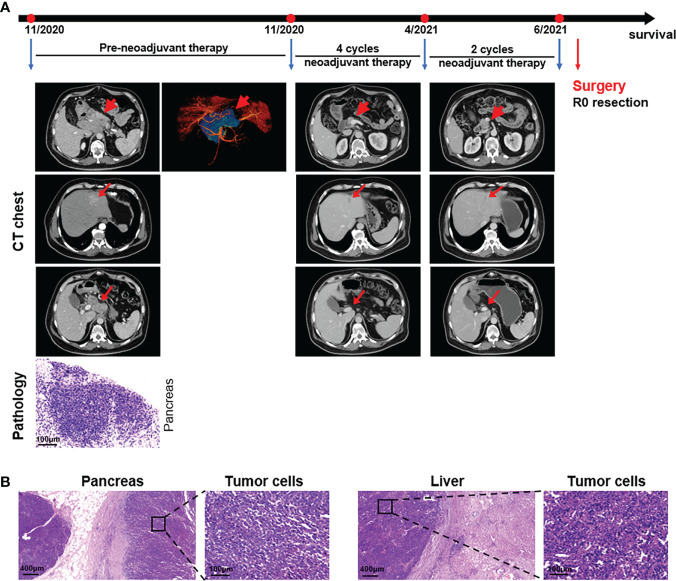
Case presentation of patient 1. **(A)** Timeline of disease status. Tumor responses shown by ECT of pre- and post-neoadjuvant therapy and pathological diagnosis of the pancreas with fine needle aspiration. **(B)** H&E staining of the isolated pancreas and liver tissues after surgery.

A 53-year-old man was a heavy smoker with a 1-year drinking history. He had an 8-year history of hepatitis B and diabetes, a 6-year history of hypertension, all well controlled. An appendectomy was performed more than 30 years ago. He had discomfort in the right upper abdomen for a month and was hospitalized on November 16, 2020. His vital signs were stable and body mass index (BMI) was 29.6 kg/m^2^. Laboratory findings were within normal limits, except the leukocyte level was 9.88×10^9^/L (normal range = 3.97-9.15), the creatinine was 43 µmol/L (normal range = 44-97), and the carbohydrate antigen 125 (CA-125) level was 36.3 IU/ml (normal range = 0-35). Upper abdomen enhanced computed tomography (ECT) demonstrated a low-density shadow 11.8×8.8 cm behind the head and body of the pancreas and a slightly low-density shadow 3.1×2.5 cm was observed in the left lateral lobe of the liver showing a “fast-in and fast-out” strengthening pattern. The portal vein was invaded and thinned by the shadow behind the head of the pancreas. Multiple enlarged lymph nodes were found in the porta hepatis and retroperitoneum; the diameter of larger one was about 4.0 cm. Biopsy revealed poorly differentiated pancreatic adenocarcinoma and no obvious cancer tissue in the liver (**Figure 1A**). The patient was diagnosed with pancreatic cancer and liver metastases (pT4N2M1).

UAPC patient preoperative downstaging therapy was first, followed by surgery. On November 21, 2020, the patient began receiving a PD-1 inhibitor, 200 mg Tislelizumab (BeiGene^®^) (Day 1), and 125 mg/m^2^ albumin-bound paclitaxel (Day 1 and Day 8) plus 1000 mg/m^2^ gemcitabine (Day 1 and Day 8) (AG) as the first-line treatment for each 21-day cycle. Low-frequency ultrasound cavitation sensitization therapy based on gemcitabine was added in the fourth cycle, and the tumor perfusion increased by 35% after the fourth treatment. At the end of six courses of treatment, the pancreatic head and body were normal on ECT, and a band-like slightly low-density shadow 1.7×0.7 cm was seen in the left lateral lobe of the liver. Several small lymph nodes were found near the head of the pancreas and retroperitoneum, and the larger one was about 0.7 cm (**Figure 1A**). However, ultrasonography revealed a hypoechoic area of 1.8×1.2 cm behind the head of the pancreas, and surgery was recommended. A pancreaticoduodenectomy and a partial hepatectomy were performed on July 1, 2021 by a multidisciplinary team (MDT). The macroscopic and microscopic histopathological examinations showed pancreas adenocarcinoma, and no tumor cells were observed on the resection margins. Tumor cells were found in the excised left liver lobe (**Figure 1B**). Seven months after resection, the patient received five cycles of 200mg PD-1 antibody (Day 1) and 60 mg/m^2^ gimeracil and oteracil potassium capsules (oral, Day 1-14) for each 21-day cycle, and his general condition was good with no clinical evidence of recurrence. The patient received a change to his chemotherapy drug regimen once the total AG levels had reached the upper limit.

### Case 2

**Figure 2:**
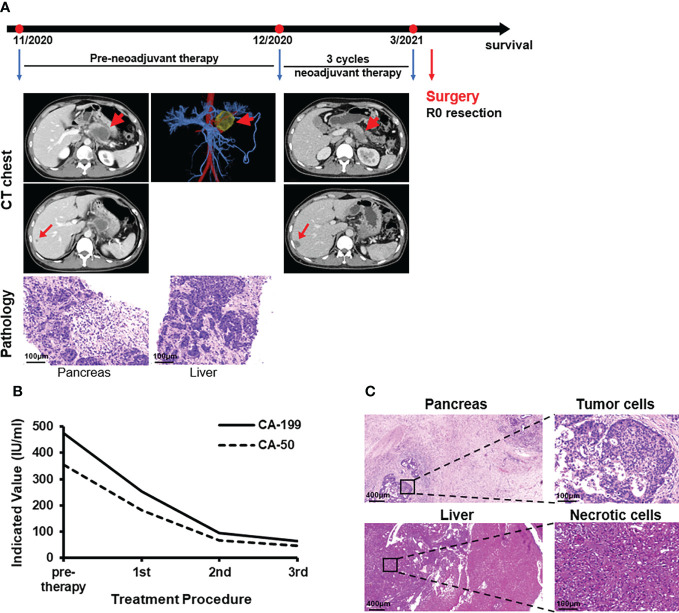
Case presentation of patient 2. **(A)** Timeline of disease status. Tumor responses shown by CT of pre- and post-neoadjuvant therapy and pathological diagnosis of pancreas and liver with fine needle aspiration. **(B)** CA-199 and CA-50 levels during treatment. 1st: receipt of the first PD-1 antibody and AG treatment. 2nd: receipt of the second treatment. 3rd: receipt of the third treatment. **(C)** H&E staining of the isolated pancreas and liver tissues after surgery.

A 43-year-old man had pain and discomfort in the upper abdomen accompanied by lower back pain for 3 months that worsened the day before hospitalization. He had no previous medical history and no abnormalities upon physical examination. Laboratory findings showed the carbohydrate antigen 199 (CA-199) level was 474 IU/ml (normal range = 0-37) and the carbohydrate antigen 50 (CA-50) level was 354 IU/ml (normal range = 0-25). ECT showed a round low-density mass of 5.2×3.9 cm in the tail of the pancreas surrounding the splenic artery and this had an unclear boundary with the coeliac trunk. The mass had invaded the splenic vein and lesser curvature of the stomach. Multiple round low-density shadows were seen in the right anterior lobe of the liver; the larger one was about 0.7×0.8 cm. Finally, the patient was diagnosed with both pancreatic adenocarcinoma and metastatic liver adenocarcinoma upon biopsy (**Figure 2A**).

PD-1 antibody and AG were considered first as a downstaging therapy for the metastatic pancreatic cancer patient. Ultrasound-guided radiofrequency ablation of liver metastases was performed with Child-Pugh grade A score during the first treatment cycle. The low-density lesions in the liver became inactive high-density lesions after ablation. At the end of three rounds of treatment, ECT showed a sheet-like low-density mass 2.4×2.3 cm in the tail of the pancreas. Multiple round low-density shadows were seen in the liver, and the larger one was about 1.8×1.3 cm (**Figure 2A**). The levels of CA-199 and CA-50 were 64.2 IU/ml and 45.2 IU/ml, respectively (**Figure 2B**). The surgical indications were already apparent and there was a high possibility of R0 resection. A distal pancreatectomy, a splenectomy, resection and radiofrequency ablation of liver metastases were carried out on March 3, 2021 after MDT. Histopathological examinations revealed pancreas adenocarcinoma with liver metastasis (pT2N2M1), and no tumor cells were observed on the resection margins (**Figure 2C**). Then five courses of PD-1 antibody plus AG were implemented and a radiofrequency ablation for intrahepatic lesions was carried out. After the total amount of AG had reached its upper limit, the patient received four cycles of 200mg PD-1 antibody (Day 1) and 80 mg/m^2^ irinotecan (Day 1) for each 21-day cycle. There was no clinical evidence of recurrence in the 12 months after resection and follow-up treatment continued.

## Discussion

Pancreatic cancer is a very difficult cancer because of its high degree of malignancy and poor prognosis, with a 5-year survival rate of less than 10% (7). The risk of pancreatic cancer is strongly associated with age, smoking, excessive BMI, genetic and family history, diabetes, or chronic pancreatitis (1). At the time of diagnosis, patients are often diagnosed with locally advanced (Stage III, 35%) or metastatic (Stage IV, 50%) pancreatic cancer, which is generally identified as UAPC according to international consensus (8). In recent years, retrospective studies have shown that patients with UAPC may benefit from surgery, and patients with systemic treatment followed by surgery have a better prognosis than those treated only with surgery. Failure to achieve R0 resection and no PNT are independent factors for poor prognosis in patients with UAPC (3, 4).

Albumin-bound paclitaxel plus gemcitabine (AG) has a good curative effect and relatively little toxicity and is a commonly used chemotherapy for pancreatic cancer. However, the rich stroma in pancreatic cancer makes it difficult for chemotherapy drugs to penetrate, resulting in few patients achieving a partial response through chemotherapy alone (9). Recently, immunotherapy has achieved remarkable results in many tumor types. Nevertheless, pancreatic cancer is generally considered to be insensitive to immunotherapy (10).

PD-1 antibody plus chemotherapy could attain better therapeutic effects, which have been seen in other tumors such as gastric cancer and lung cancer (11, 12). The 2017 American Society of Clinical Oncology annual meeting reported that a phase I study of advanced pancreatic cancer patients without systemic treatment showed that the objective response rate reached 50% following nivolumab combined with AG. Although the sample size was small, this suggested that immunotherapy plus chemotherapy may have clinical benefits. Chemotherapy stimulates neoantigens (13), turning pancreatic carcinoma from a “cold tumor” to a “hot tumor”, and enhancing the sensitivity of pancreatic tumors to immunotherapy, thus contributing to the anti-tumor effect. However, a recent report showed that the objective response rate (ORR) and disease control rate (DCR) of nivolumab plus AG and camrelizumab combined with AG for pancreatic cancer patients were different, suggesting that varying responses to the same therapy but different antibody (14).

Tislelizumab is a humanized recombinant immunoglobulin G4 monoclonal antibody against PD-1, which blocks the PD-1/PD-L1 signaling pathway and releases the “brakes” of the immune system. It is currently the only PD-1 monoclonal antibody successfully modified on the Fc, which minimizes binding to the Fcγ receptor (FcγR) on the surface of macrophages and avoids the consumption of T cells caused by antibody-dependent cell-mediated phagocytosis (ADCP) (15). Tislelizumab has low toxicity and a long half-life and has been approved in China for relapsed or refractory classic Hodgkin’s lymphoma, locally advanced or metastatic urothelial carcinoma, and advanced non-small cell lung cancer. The case 1 patient with multiple previous medical histories was in good condition during preoperative neoadjuvant therapy, the adverse effects were tolerable, and no serious adverse event occurred. The case 2 patient had no side effects.

Predictive markers are critical for improving treatment outcomes. PD-1 antibodies have received FDA approval for treating deficiency mismatch repair/high microsatellite instability (dMMR/MSI-H) or non-high tumor mutation burden (TMB-H) tumors. However, the clinical benefit of immune checkpoint inhibitors in treating pancreatic cancer remains poorly understood and is unable to effectively predict those patients most likely to respond to ICI treatment. The two cases in this report both had MSS, but showed extremely high sensitivity to PD-1 antibody (**Table 1**). PD-LI expression is one of the most effective predictors of current PD-1/PD-L1 therapy. While PD-L1 is a powerful predictive marker for non-small cell lung cancer (NSCLC) treatment response, there is insufficient evidence to support PD-L1 as a marker for pancreatic cancer treatment response. Case 1 (CPS = 85) and case 2 (CPS = 3) were both PD-L1 positive and reached strongly to PD-1 antibody (**Table 1**). Additional cases will need to be assessed to further evaluate outcomes, however, the results from this study do suggest that multiple indicators should be considered when formulating immunotherapy regimens.

**Table 1:** The molecular background of two cases.

	PD-L1	Microsatellite	Mutant Gene	
			I	II
Case 1	Positive	MSS	NA	*TP53* *CCND1* *FGF19*
Case 2	Positive	MSS	NA	*KRAS* *BRCA2* *FGF19* *MYC*

MSS, microsatellite stability; I, mutations with clear clinical significance corresponding to drug sensitivity grade 1; II, mutations with potential clinical significance corresponding to drug susceptibility grade 2-4; NA, no mutation detected.

Our study contributes to the general knowledge base about how best to treat patients with UAPC. Two patients herein underwent PNT (PD-1 antibody immunotherapy combined with chemotherapy) and ultimately achieved R0 resection. However, there were limitations such as a lack of control cases and a short observation period, which could not show postoperative improvement in overall survival. In the future, larger samples in clinical trial are needed to verify the clinical efficacy of our neoadjuvant treatment model in UAPC.

## Data availability statement

The original contributions presented in the study are included in the article supplementary material. Further inquiries can be directed to the corresponding author.

## Ethics statement

Written informed consent was obtained from all patients for the publication of any potentially identifiable images or data included in this article.

## Author contributions

LS and PL provided patient information. JF collected and analyzed the data. CZ and QB took care of the patients. XN took pathology photos. ZY and YK participated in treatment plans. TZ, BX, and JD helped with revising the manuscript. LS, PL, and HX wrote the paper. All authors contributed to the article and approved the submitted version.

## Acknowledgments

We thank Professor Yue Shuqiang of the Department of Hepatobiliary and Pancreatic Surgery, Xijing Hospital, Air Force Medical University, for providing surgical guidance. We thank the patients for allowing us to share their medical history and clinical course.

## Conflict of interest

The authors declare that the research was conducted in the absence of any commercial or financial relationships that could be construed as a potential conflict of interest.

## Publisher’s note

All claims expressed in this article are solely those of the authors and do not necessarily represent those of their affiliated organizations, or those of the publisher, the editors and the reviewers. Any product that may be evaluated in this article, or claim that may be made by its manufacturer, is not guaranteed or endorsed by the publisher.
